# Supplementary material for: Sparse and Compositionally Robust Inference of Microbial Ecological Networks
Source: PLoS Comput Biol. 2015 May 7;11(5):e1004226. doi: 10.1371/journal.pcbi.1004226 (PMC4423992; doi:10.1371/journal.pcbi.1004226)
Supplement: S1 Text — A discussion of the methods used to simulate biologically-plausible correlated count data, and some results comparing different generative distributions. (PDF) [file pcbi.1004226.s001.pdf]

A Appendix

A.1 Comparative marginal fits to American Gut Project count data

We considered five common distributions for modeling OTU count data in SPIEC-EASI's synthetic data generator. As as target count data we used rounded common-scale normalized data from the American Gut Project (AGP). After filtering, a total of $p = 205$ different OTU counts $w_i \in \mathbb{N}_0^n$ of sample size $n = 558$ are available for marginal fitting. We fit the parameters of each distributional model to each OTU count vector w_i independently using maximum likelihood (ML) estimation.

A.1.1 Common count distributions

We considered the following five common distributions to model univariate count data:

Log-normal. The log-normal distribution is a continuous distribution of a random variable U whose logarithm is normally distributed, $U \sim \ln \mathcal{N}(\mu, \sigma)$, where μ and σ are the mean and standard deviation of $\ln(U)$, respectively. Its probability density function is given by

$$P(U = u) = \frac{1}{u\sigma\sqrt{2\pi}} e^{-\frac{(\ln u - \mu)^2}{2\sigma^2}} , \tag{1}$$

To model discrete counts, the continuous values can be discretized by rounding to the nearest integer.

Poisson. The Poisson distribution models a given number of discrete "events" occurring in a fixed interval (a of an ecosystem). In ecology, a Poisson distributed random variable $U \sim \ln \mathrm{Pois}(\lambda)$ describes the number of occurrences of a species across different ecosystem samples. The probability mass function is given by:

$$P(U = u) = \frac{\lambda^u e^{-\lambda}}{u!} , \tag{2}$$

where λ is the Poisson 'rate' parameter that determines both mean and variance.

Zero-inflated Poisson. Zero-inflated models account for an excess of zero counts in real data that cannot be handled by a single component model. In ecology, count data may be skewed toward zero due to a preponderance of counts that fall below the sampling depth. The zero-inflated Poisson (ziP) model incorporates an additional term to account for this feature. The random variable reads:

$$U \sim \begin{cases} 0 & \text{with prob. } \phi \\ \mathrm{Pois}(\lambda) & \text{with prob. } 1 - \phi \end{cases}$$

and its mass function is given by:

$$P(U = 0) = \phi + (1 - \phi)e^{-\lambda} \tag{3}$$

$$P(U = u) = (1 - \phi)\frac{\lambda^u e^{-\lambda}}{u!} \tag{4}$$

where ϕ is the probability of obtaining an excess zero and λ is the Poisson rate parameter as above. When $\phi = 0$, the ziP distribution reduces to the Poisson distribution.

Negative Binomial. The negative binomial (NB) distribution arises as a hierarchical mixture of Poisson distributions that can model the variability from multiple sources (such as, e.g., biological replication and library preparation) [56]. The NB distribution is thus more appropriate for handling overdispersion in the data. We assume the means of the Poisson variables to be Gamma-distributed random variables with shape hyper-parameter r and scale $\theta = p/(1 - p)$. First, a random Poisson mean λ is sampled from a Gamma

distribution $\Gamma(r, \theta)$, and then a random variable u from $\text{Pois}(\lambda)$. The compact form of the mass distribution of a discrete NB random variable $U \sim \text{NB}(\text{r}; \text{p})$ then reads:

$$P(U = u) = \frac{\Gamma(r+u)}{u!\,\Gamma(r)} p^u (1-p)^r \,. \tag{5}$$

Here, overdispersion is controlled by the parameter r. The NB model collapses to the Poisson model for $r \to \infty$.

Zero-inflated Negative Binomial Similar to the ziP model, the zero-inflated NB (ziNB) distribution can better account for excess zeros in the observed data. The standard NB distribution is augmented by a zero-inflation term. We denote a ziNB random variable by:

$$U \sim \begin{cases} 0 & \text{with prob. } \phi \\ \text{NB}(r; p) & \text{with prob. } 1 - \phi \,, \end{cases} \tag{6}$$

where ϕ is the probability of obtaining an excess zero. The corresponding ziNB mass function reads:

$$P(U = 0) \;=\; \phi + (1-\phi) p^u \tag{7}$$

$$P(U = u) \;=\; (1-\phi) \frac{\Gamma(r+u)}{u!\,\Gamma(r)} p^u (1-p)^r \,. \tag{8}$$

For $\phi = 0$ the ziNB model reduces to the NB distribution.

A.1.2 Goodness-of-fit to the AGP data

The AGP data was normalized using common-scale normalization, and fractional counts were rounded. We fit the count data to the five different statistical models and drew synthetic data (under null correlation between OTU marginals) from these distributions with the ML fitted parameters. Figure A.1 shows QQ plots of synthetic and real data for the five different distributions.

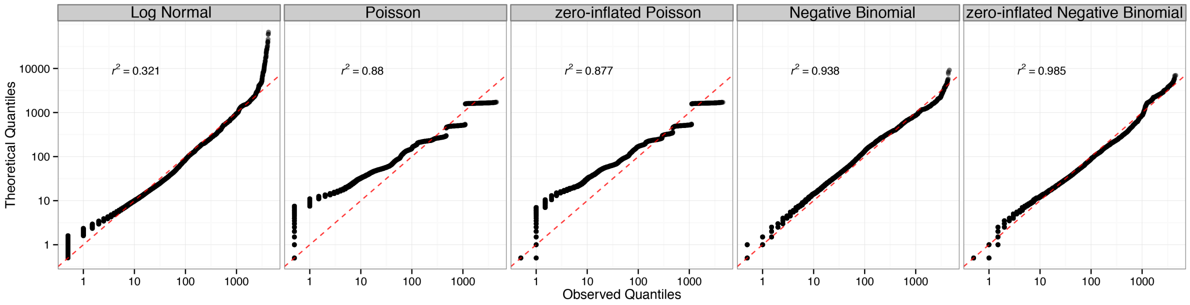

Figure A.1: QQ plots of AGP data fit to five different distributions (shown on a log-log scale for better visibility). The r^2 values correspond to the unscaled quantile-quantile relationships.

The ziNB distribution ($r^2 = 0.985$) is the only model that can accurately model both tails of the OTU data. We thus use the ziNB as the standard setting in SPIEC-EASI's data generation model.

References

[56] McMurdie PJ, Holmes S (2014) Waste Not, Want Not: Why Rarefying Microbiome Data Is Inadmissible. PLoS Computational Biology 10: e1003531.
