# Supplementary material for: Sparse and Compositionally Robust Inference of Microbial Ecological Networks
Source: PLoS Comput Biol. 2015 May 7;11(5):e1004226. doi: 10.1371/journal.pcbi.1004226 (PMC4423992; doi:10.1371/journal.pcbi.1004226)
Supplement: S1 Table — Table to compare some of the features of SPIEC-EASI, SparCC, CCREPE and Pearson’s correlation coefficient. (PDF) [file pcbi.1004226.s002.pdf]

	SPIEC EASI	SparCC	CCREPE	Pearson
Underlying Metric	Conditional Independence	Correlation	Correlation	Correlation
Compositional Correction	Yes	Yes	Yes	No
Aitchison Measure	Inverse of clr covariance matrix	Aitchison Variation	-	-
Network Assumptions	Network sparsity	Average correlation is zero	-	-
