# Supplementary figures and images for: Sparse and Compositionally Robust Inference of Microbial Ecological Networks
Source: PLoS Comput Biol. 2015 May 7;11(5):e1004226. doi: 10.1371/journal.pcbi.1004226 (PMC4423992; doi:10.1371/journal.pcbi.1004226)

**a**

Band

Cluster

Scale-free

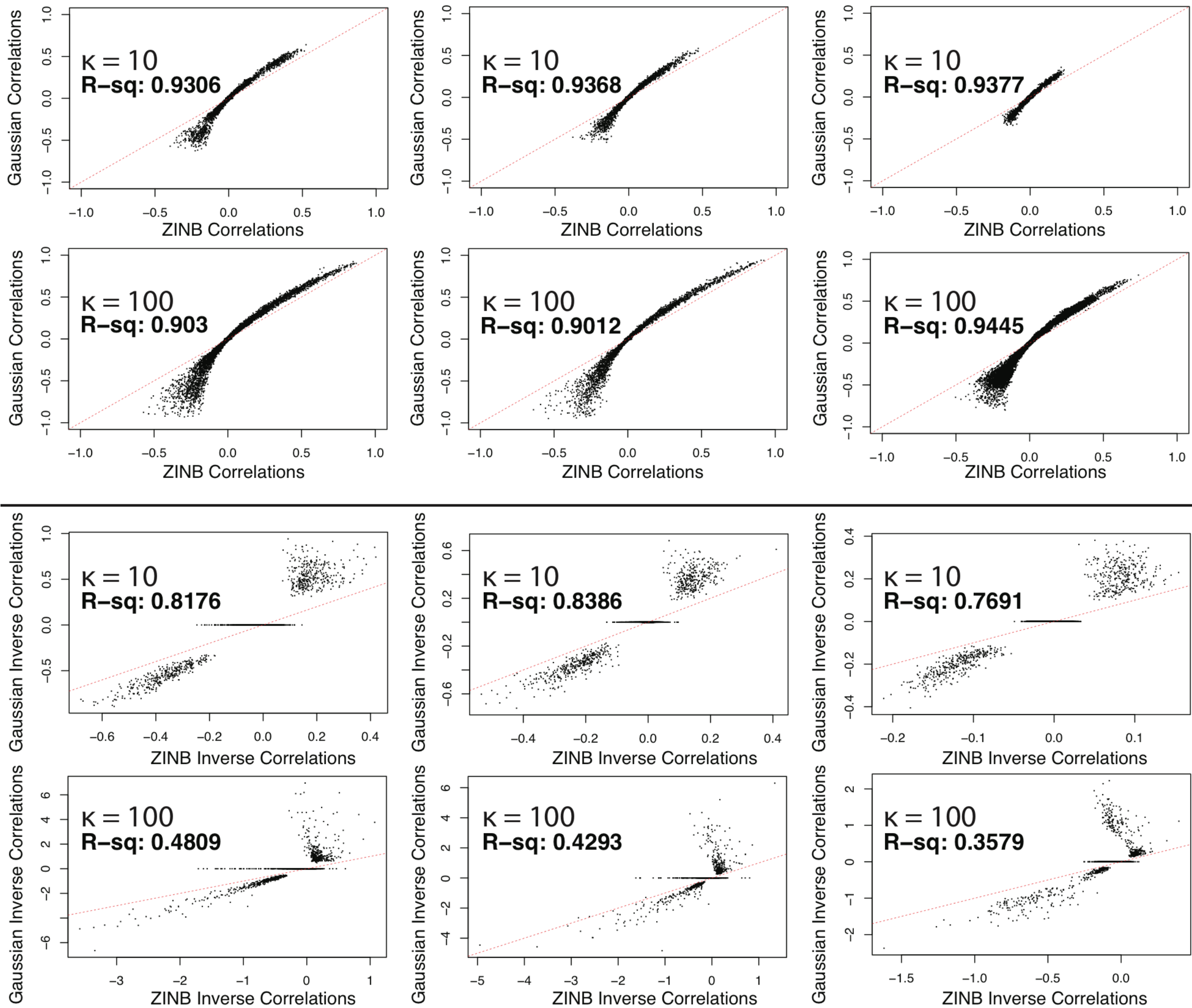**b**

Band

Cluster

Scale-free

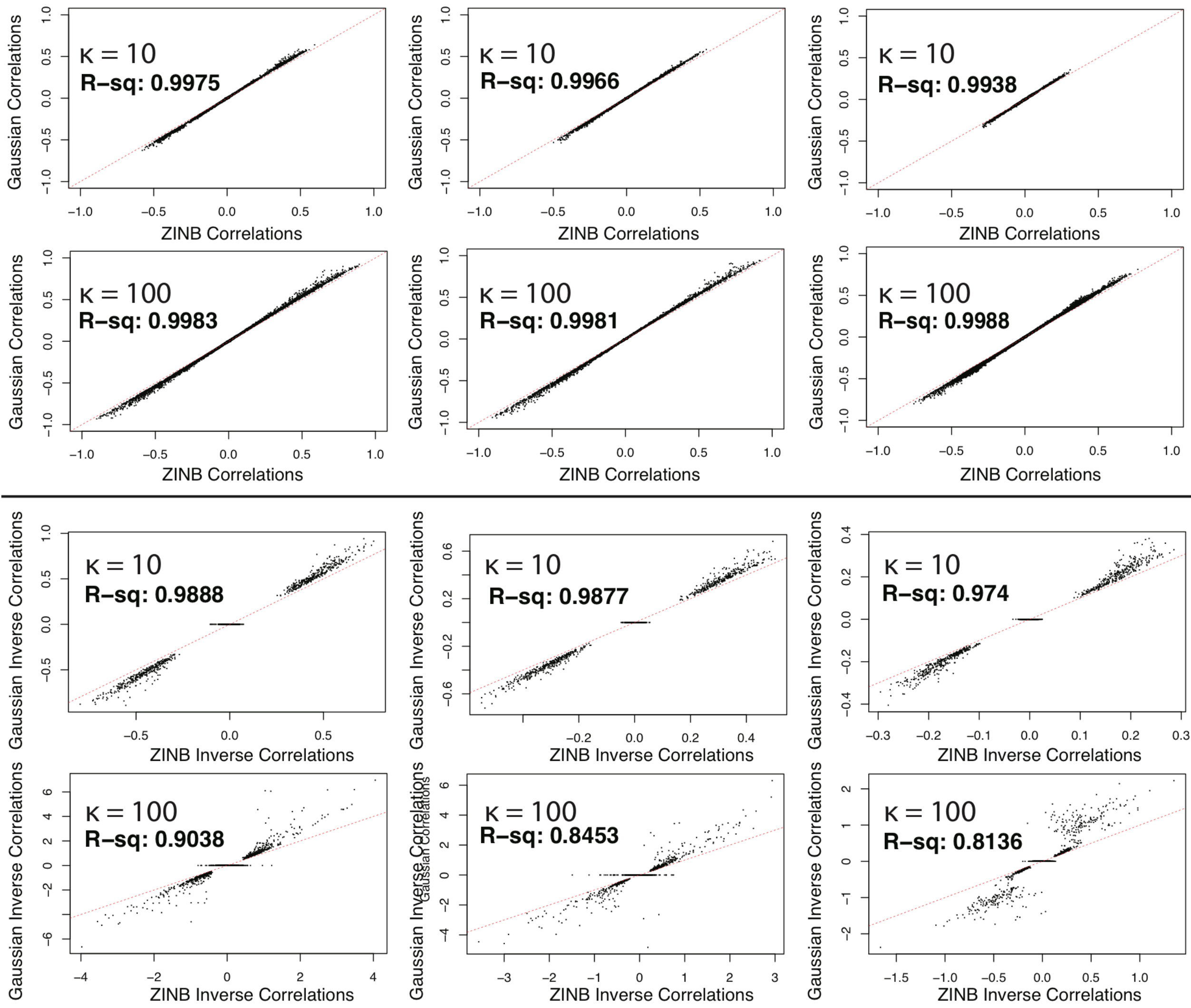

Supplement: S1 Fig — The recovery of empirical Pearson correlations generated from the NORTA process, using zero-inflated Negative Binomial as a model (x-axis) verses the input multivariate Normal empirical correlations (upper panels) or inverse correlations (lower panels) on untransformed counts (a) or log-transformed counts (b). Simulated data are with p = 205 OTUs, n = 20,000 samples with 10 replicates on each plot. (PDF) [file pcbi.1004226.s003.pdf]

**Band**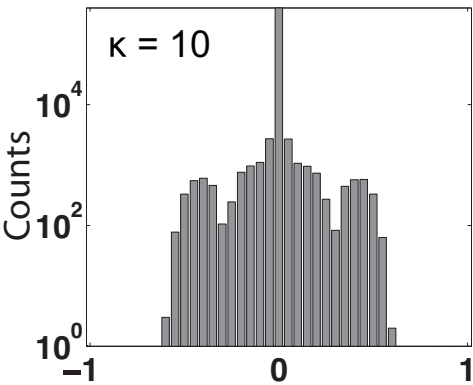**Cluster**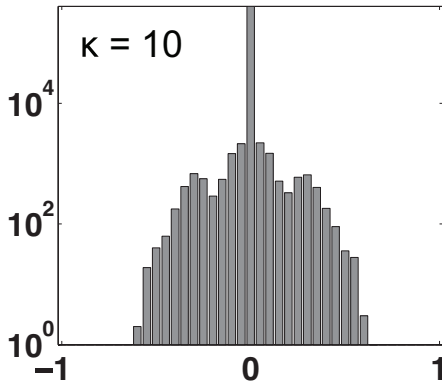**Scale-free**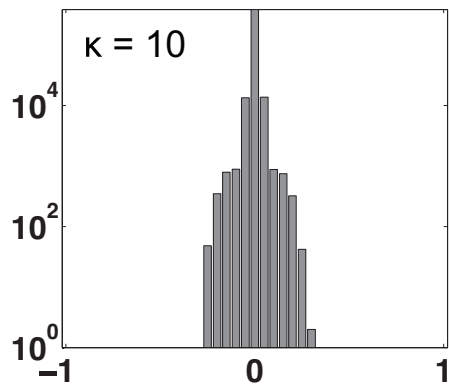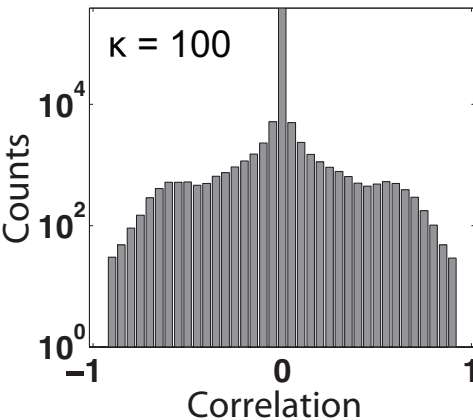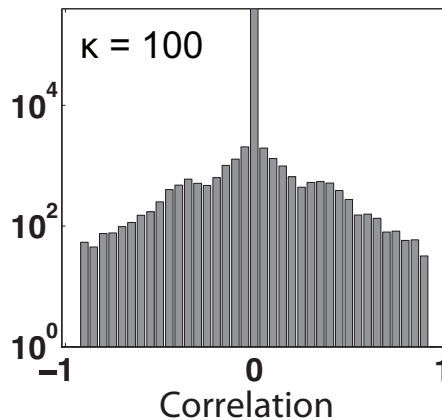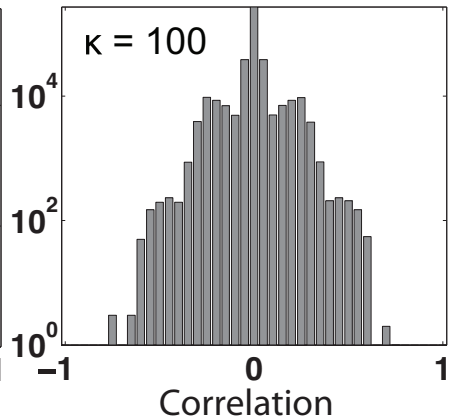

Supplement: S3 Fig — The condition of a Precision matrix is the ratio of the largest to smallest eigenvalue/singular value. The relationship between condition number and correlation distribution for the synthetic networks; increasing condition number corresponds to increasing the strength of correlations in the network. (PDF) [file pcbi.1004226.s005.pdf]

**Betweenness (Band, S-E(MB),  $D_{KL} = 0.82$ )**

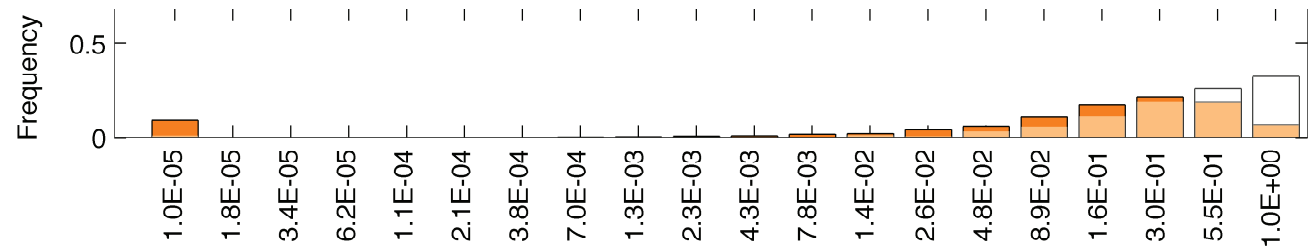

**Betweenness (Cluster, S-E(MB),  $D_{KL} = 0$ )**

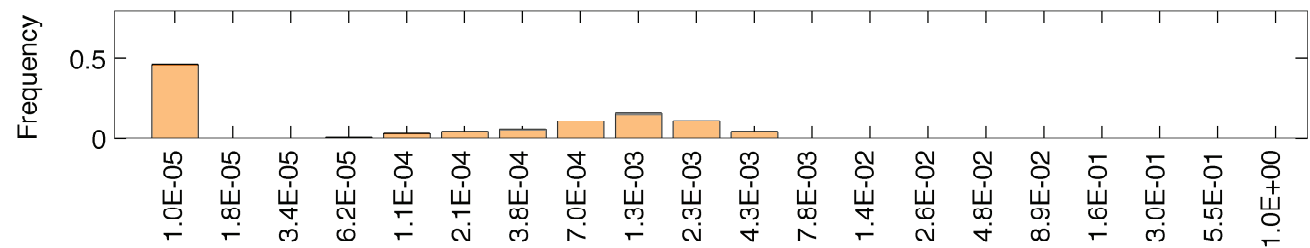

**Betweenness (Scale-free, S-E(MB),  $D_{KL} = 0.73$ )**

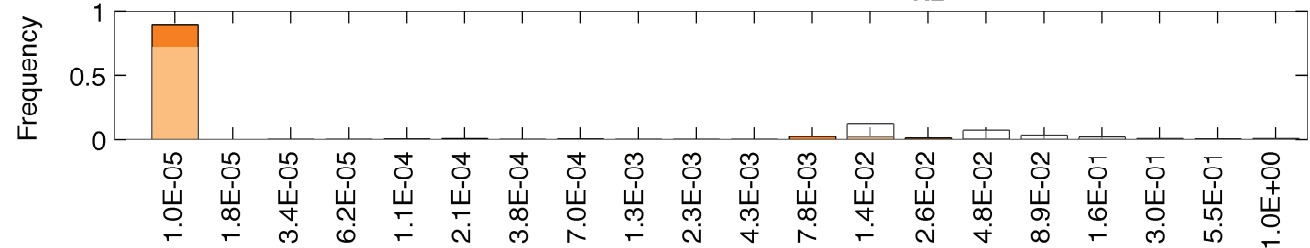

Supplement: S4 Fig — Examples of betweenness centrality distributions for each network type (in white) overlayed with the distribution predicted by S-E(MB) (in orange) for κ = 100, n = 1360 samples, p = 205 OTUs. (PDF) [file pcbi.1004226.s006.pdf]

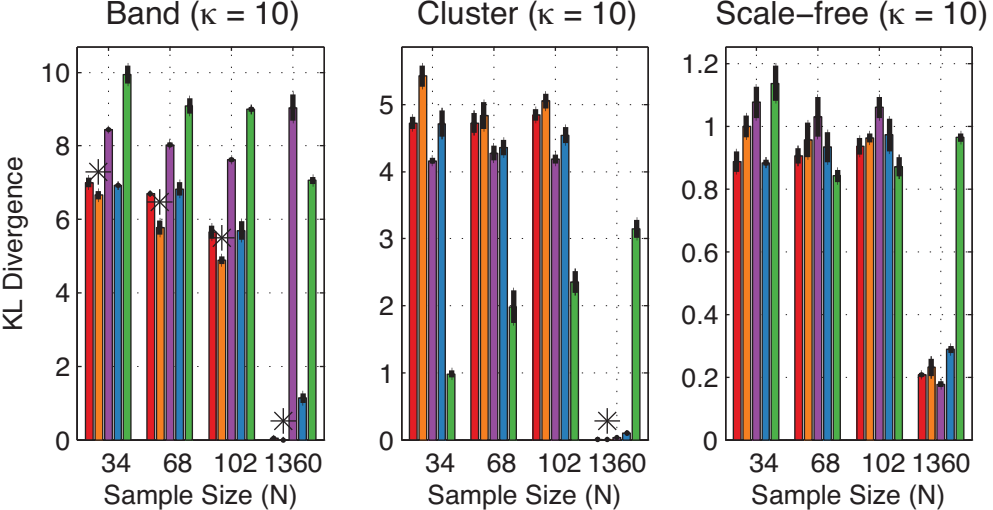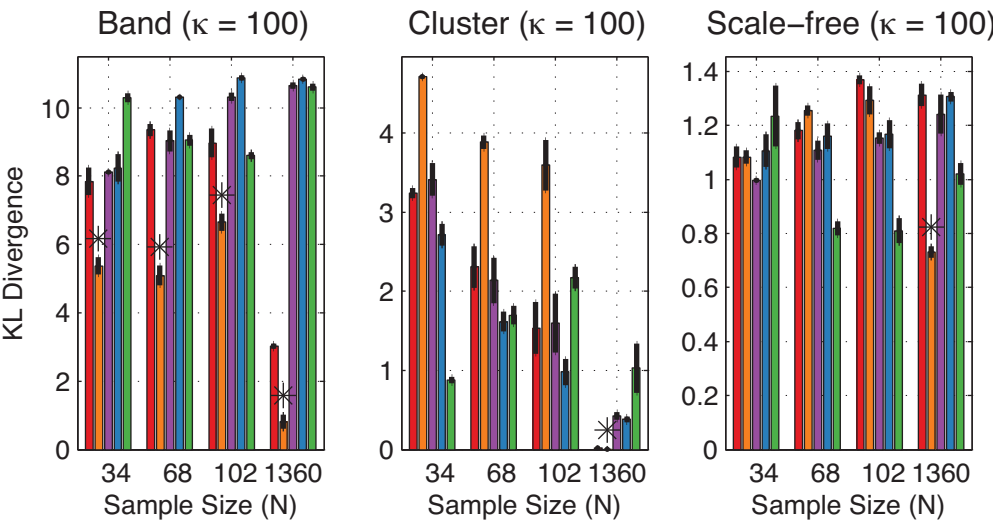

Supplement: S5 Fig — Performance results for betweenness centrality (red = S-E(glasso) orange = S-E(MB), purple = SparCC, blue = CCREPE, green = Pearson). Bars represent the average KL-divergence over three independent sets of synthetic datasets (7 datasets per set); error bars represent standard error. Asterisks indicate that an S-E method had siginificantly better recovery of the true betweenness centrality distributions (p < 0.05 for one-sided T tests in comparison to each control method). (PDF) [file pcbi.1004226.s007.pdf]

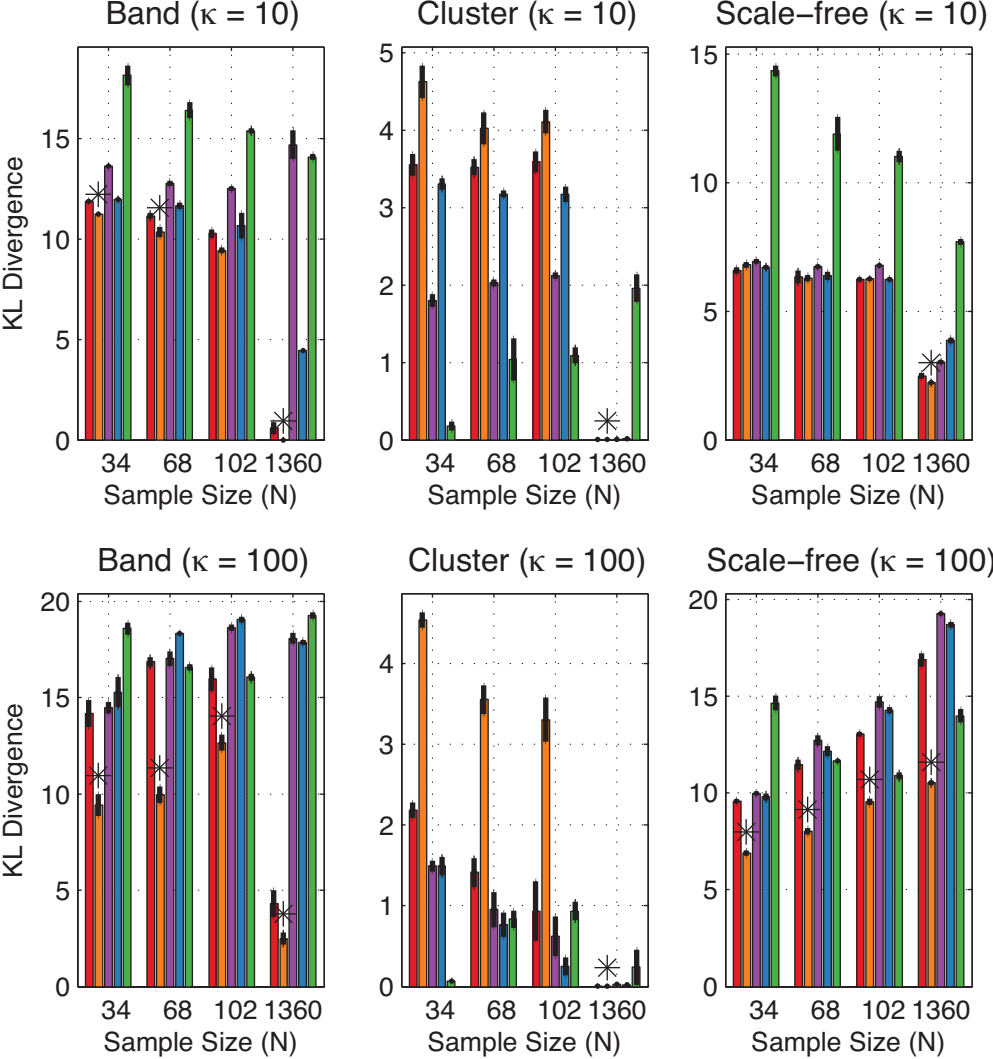

Supplement: S7 Fig — Performance results for geodesic distance distributions (red = S-E(glasso), orange = S-E(MB), purple = SparCC, blue = CCREPE, green = Pearson). Bars represent the average KL-divergence over three independent sets of synthetic datasets (7 datasets per set); error bars represent standard error. Asterisks indicate that an S-E method had significantly better recovery of the true geodesic distance distributions (p < 0.05 for one-sided T tests in comparison to each control method). (PDF) [file pcbi.1004226.s009.pdf]

**Cluster Sizes (Band, S-E(MB),  $D_{KL} = 2.54$ )**

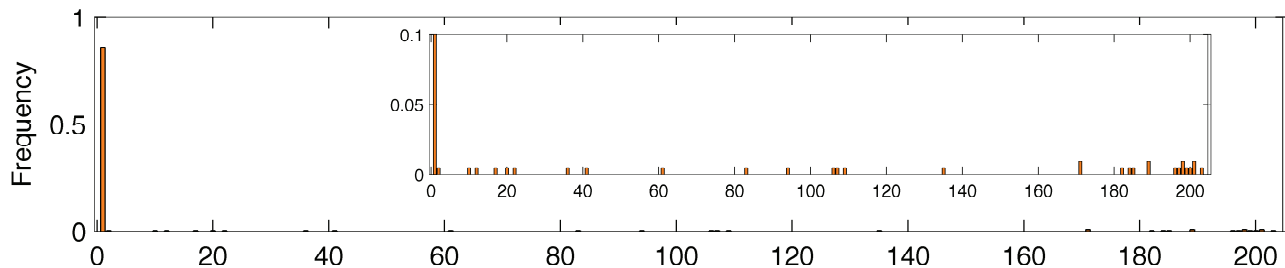

**Cluster Sizes (Cluster, S-E(MB),  $D_{KL} = 0$ )**

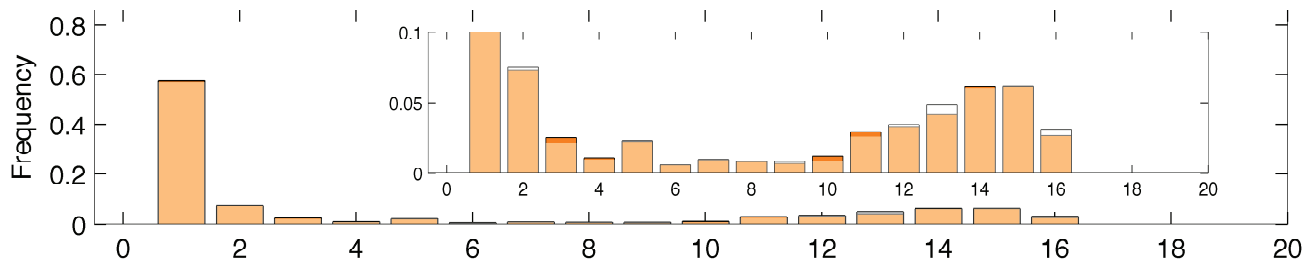

**Cluster Sizes (Scale-free, S-E(MB),  $D_{KL} = 6.79$ )**

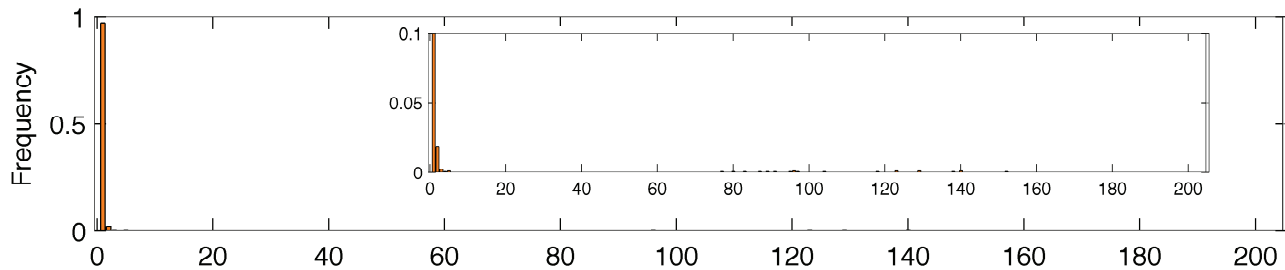

Supplement: S8 Fig — Examples of cluster size distributions for each network type (in white) overlayed with the distribution predicted by S-E(MB) (in orange), for κ = 100, n = 1360 samples, p = 205 OTUs. (PDF) [file pcbi.1004226.s010.pdf]

Band ( $\kappa = 10$ )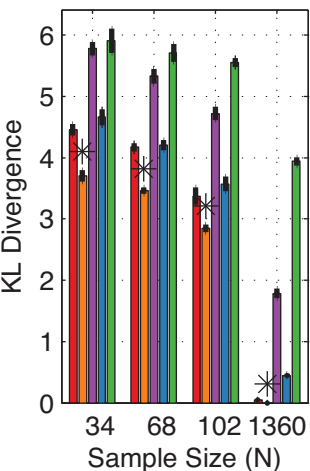Cluster ( $\kappa = 10$ )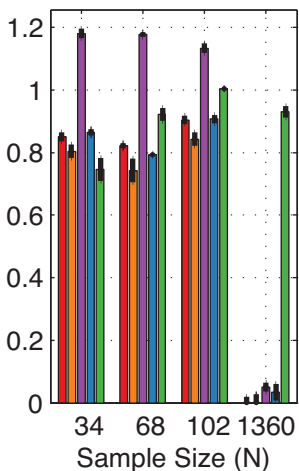Scale-free ( $\kappa = 10$ )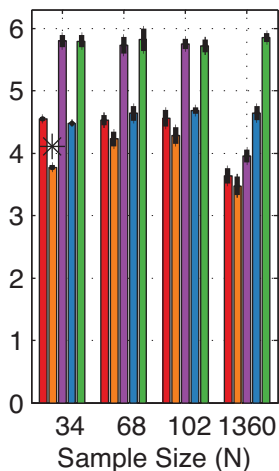Band ( $\kappa = 100$ )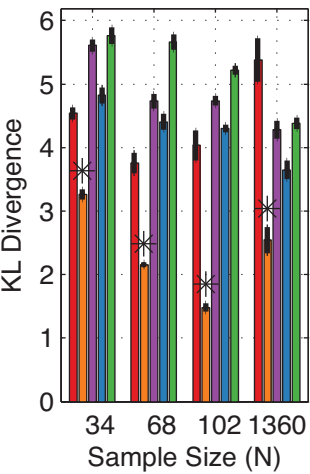Cluster ( $\kappa = 100$ )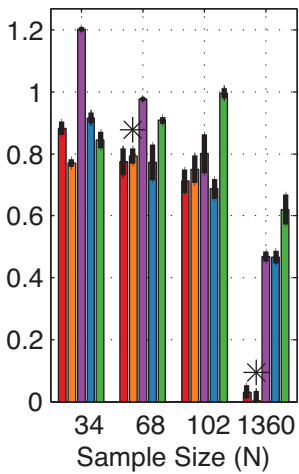Scale-free ( $\kappa = 100$ )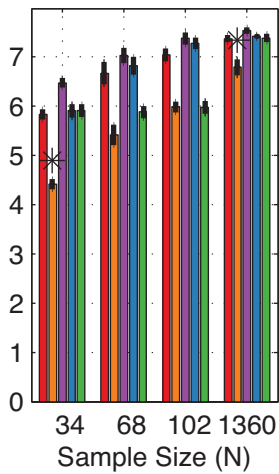

Supplement: S9 Fig — Performance results for cluster size distributions (red = S-E(glasso), orange = S-E(MB), purple = SparCC, blue = CCREPE, green = Pearson). Bars represent the average KL-divergence over three independent sets of synthetic datasets (7 datasets per set); error bars represent standard error. Asterisks indicate that an S-E method had significantly better recovery of the true cluster size distributions (P < 0.05 for one-sided T tests in comparison to each control method). (PDF) [file pcbi.1004226.s011.pdf]

# True Correlation

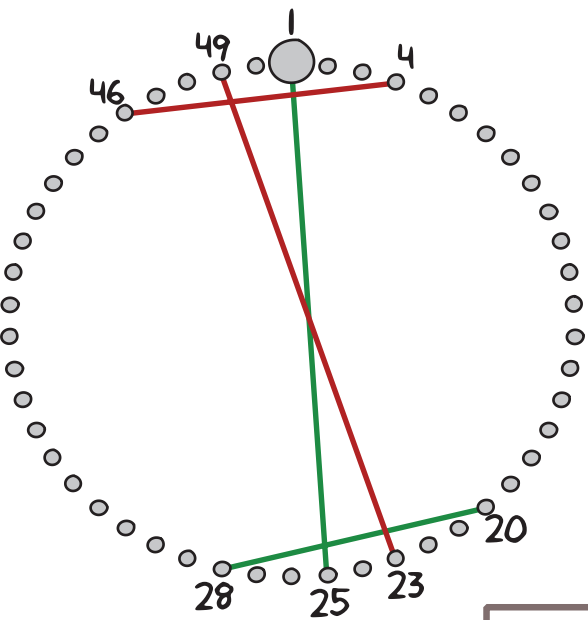

# SparCC

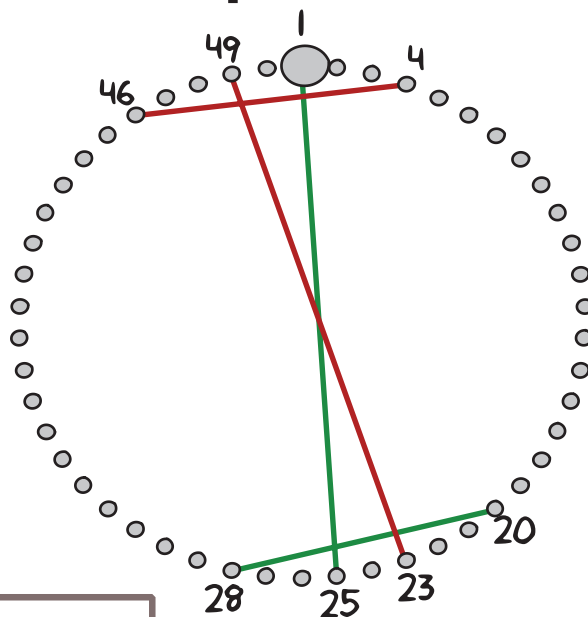

# S-E.MB

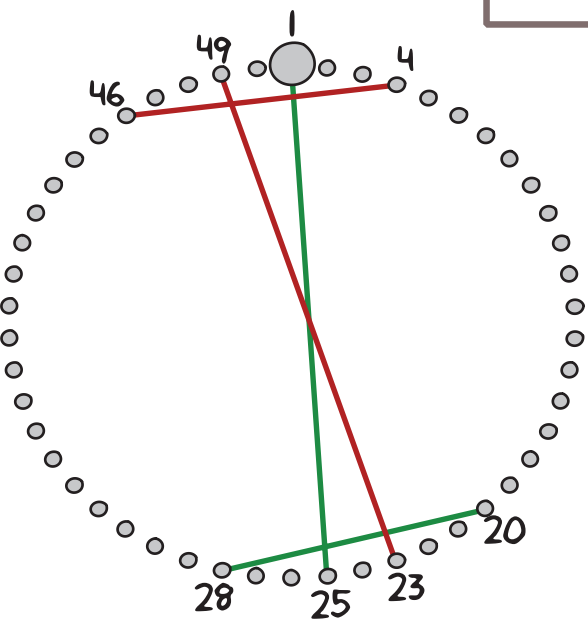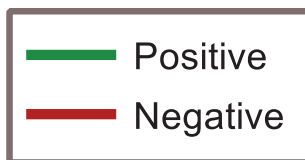

# CCREPE

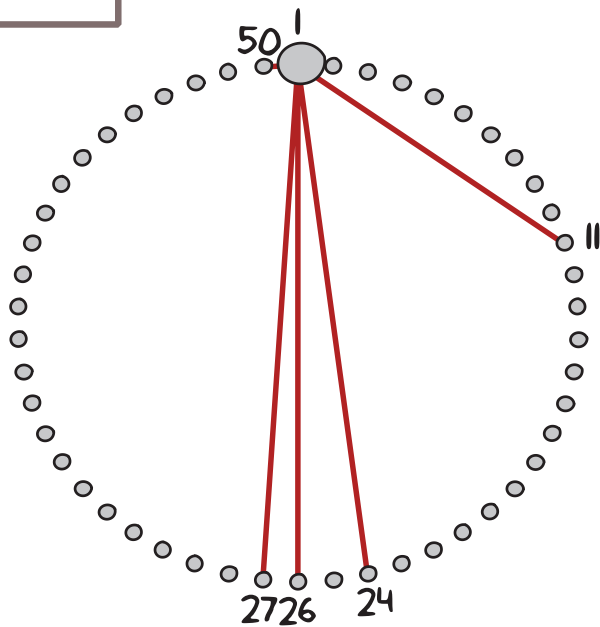

Supplement: S10 Fig — Using the example data provided by the SparCC package, we inferred networks from SparCC (threshold correlation at ±.35), S-E(MB) and CCREPE (thresholding q-value at 5 * 10−19). In this setting, SparCC and SPIEC-EASI correctly recover four true edges, including the association sign. (PDF) [file pcbi.1004226.s012.pdf]

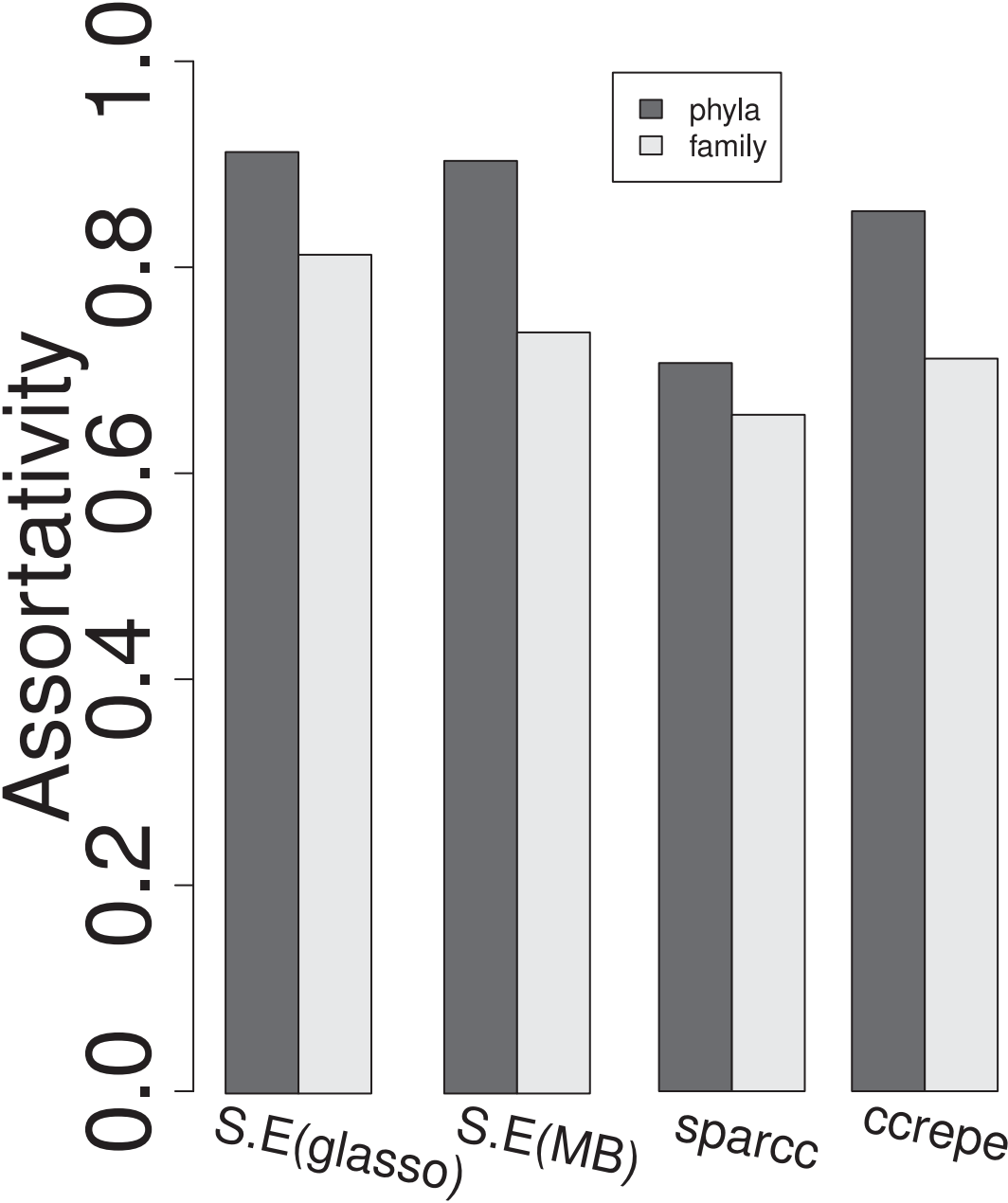

Supplement: S11 Fig — Network assortativity coefficients at the Phyla and Family level for each of the four inference methods. Assortativity is a measure of the tendency for nodes to be connected with nodes of the same taxonomic class. (PDF) [file pcbi.1004226.s013.pdf]
