# Supplementary material for: Sparse and Compositionally Robust Inference of Microbial Ecological Networks
Source: PLoS Comput Biol. 2015 May 7;11(5):e1004226. doi: 10.1371/journal.pcbi.1004226 (PMC4423992; doi:10.1371/journal.pcbi.1004226)
Supplement: S2 Fig — Visual comparison of American Gut Project data with synthetically generated datasets. Heatmaps show log10(counts). For all network types (band, graph, scale-free), synthetic datasets are consistent with real datasets in terms of number of OTUs, number of samples, and OTU count abundances across samples. (PDF) [file pcbi.1004226.s004.pdf]

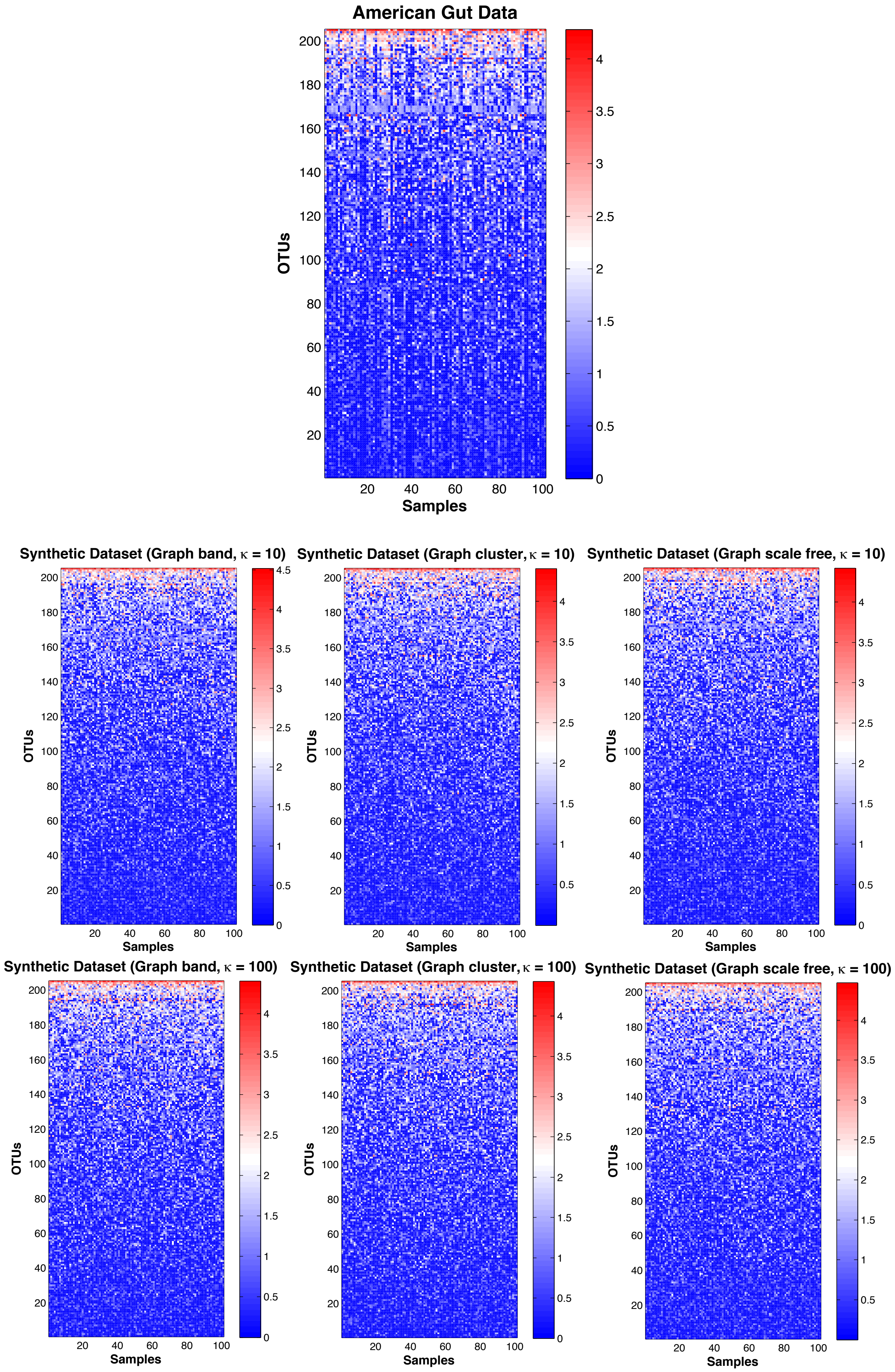
American Gut Data
OTUs
Samples
200
180
160
140
120
100
80
60
40
20
20
40
60
80
100
0
0.5
1
1.5
2
2.5
3
3.5
4
Synthetic Dataset (Graph band, κ = 10)
Synthetic Dataset (Graph cluster, κ = 10)
Synthetic Dataset (Graph scale free, κ = 10)
Synthetic Dataset (Graph band, κ = 100)
Synthetic Dataset (Graph cluster, κ = 100)
Synthetic Dataset (Graph scale free, κ = 100)
4.5
