# Supplementary material for: Sparse and Compositionally Robust Inference of Microbial Ecological Networks
Source: PLoS Comput Biol. 2015 May 7;11(5):e1004226. doi: 10.1371/journal.pcbi.1004226 (PMC4423992; doi:10.1371/journal.pcbi.1004226)
Supplement: S6 Fig — Examples of geodesic distance distributions for each network type (in white) overlaid with the distribution predicted by S-E(MB) (in orange) for κ = 100, n = 1360 samples, p = 205 OTUs (PDF) [file pcbi.1004226.s008.pdf]

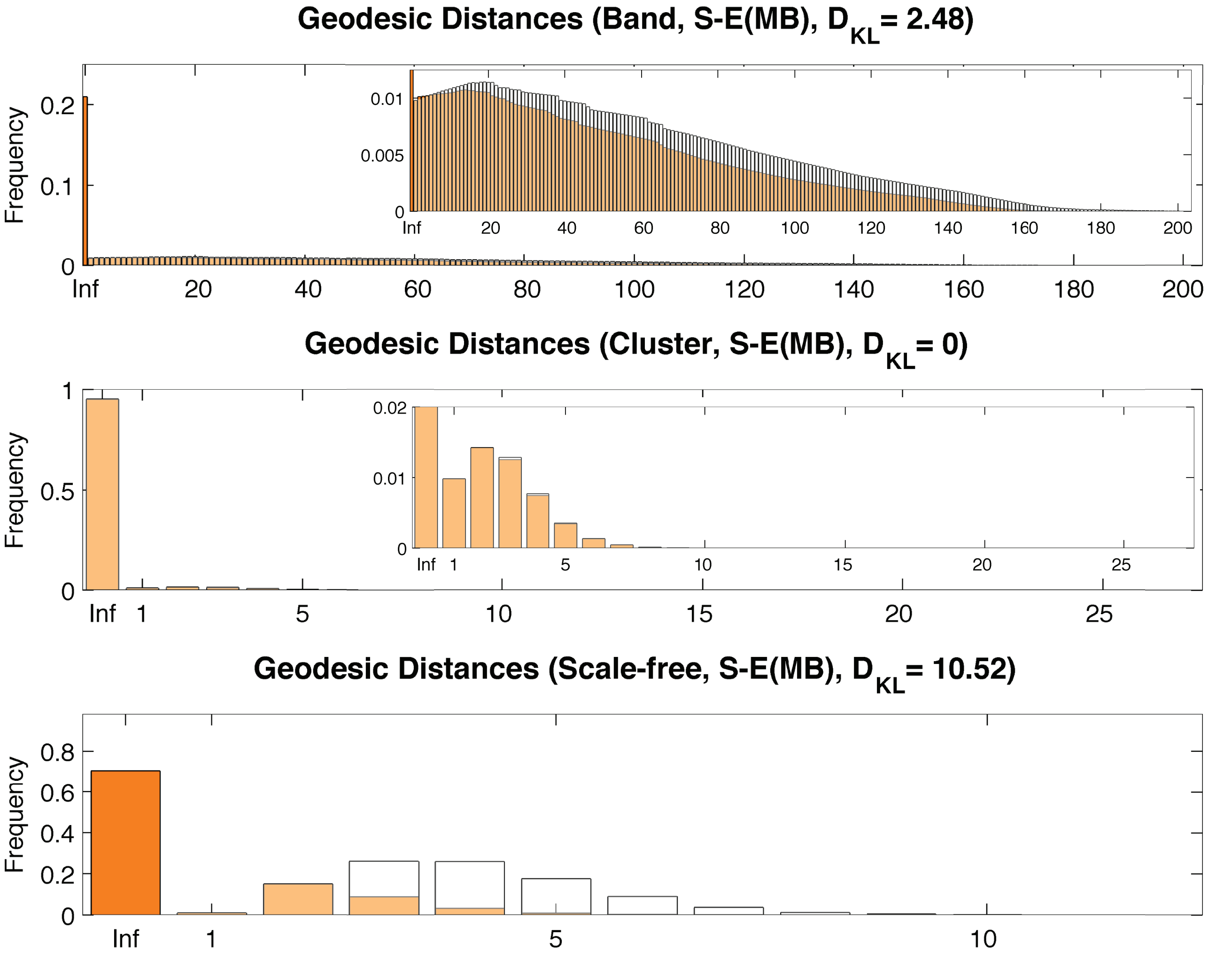
Geodesic Distances (Band, S-E(MB), D_KL= 2.48)
Frequency
0.2
0.1
0
Inf
20
40
60
80
100
120
140
160
180
200
0.01
0.005
Geodesic Distances (Cluster, S-E(MB), D_KL= 0)
1
0.5
0.02
Inf 1
5
10
15
20
25
Geodesic Distances (Scale-free, S-E(MB), D_KL= 10.52)
0.8
0.6
0.4
